# Pulse Wave Variation during the Menstrual Cycle in Women with Menstrual Pain

**DOI:** 10.1155/2016/1083208

**Published:** 2016-08-07

**Authors:** Soo Hyung Jeon, Kyu Kon Kim, In Seon Lee, Yong Tae Lee, Gyeong Cheol Kim, Gyoo Yong Chi, Hye Sook Cho, Hee Jung Kang, Jong Won Kim

**Affiliations:** ^1^Department of Sasang Constitutional Medicine, College of Korean Medicine, Dong-Eui University, Yangjeong-ro 62, Busanjin-gu, Busan 47227, Republic of Korea; ^2^Department of Data Information Science, Dong-Eui University, Eomgwang-ro 176, Busanjin-gu, Busan 47340, Republic of Korea; ^3^Department of OB & GY, College of Korean Medicine, Dong-Eui University, Yangjeong-ro 62, Busanjin-gu, Busan 47227, Republic of Korea; ^4^Department of Physiology, College of Korean Medicine, Dong-Eui University, Yangjeong-ro 62, Busanjin-gu, Busan 47227, Republic of Korea; ^5^Department of Diagnostics, College of Korean Medicine, Dong-Eui University, Yangjeong-ro 62, Busanjin-gu, Busan 47227, Republic of Korea; ^6^Department of Pathology, College of Korean Medicine, Dong-Eui University, Yangjeong-ro 62, Busanjin-gu, Busan 47227, Republic of Korea; ^7^Daeyomedi Co., Ltd., 601 Gyeonggi Technopark, Haean-ro 705, Sangnok-gu, Ansan-si, Gyeonggi-do 15588, Republic of Korea

## Abstract

*Objective*. This study is performed to obtain objective diagnostic indicators associated with menstrual pain using pulse wave analysis.* Methods*. Using a pulse diagnostic device, we measured the pulse waves of 541 women aged between 19 and 30 years, placed in either an experimental group with menstrual pain (*n* = 329) or a control group with little or no menstrual pain (*n* = 212). Measurements were taken during both the menstrual and nonmenstrual periods, and comparative analysis was performed.* Results*. During the nonmenstrual period, the experimental group showed a significantly higher value in the left radial artery for the radial augmentation index (RAI) (*p* = 0.050) but significantly lower values for pulse wave energy (*p* = 0.021) and time to first peak from baseline (*T*1) (*p* = 0.035) in the right radial artery. During the menstrual period, the experimental group showed significantly lower values in the left radial artery for cardiac diastole and pulse wave area during diastole and significantly higher values for pulse wave area during systole, ratio of systolic phase to the full heartbeat, and systolic-diastolic ratio.* Conclusion*. We obtained indicators of menstrual pain in women during the menstrual period, including prolonged systolic and shortened diastolic phases, increases in pulse wave energy and area of representative pulse wave, and increased blood vessel resistance.

## 1. Introduction

The pulse wave is one of the four major diagnostic methods (四診) used in traditional Korean medicine (TKM) and is recognized as an objective method in standard clinical practice guidelines [1, 2]. In recent years, diverse research studies have investigated scientific approaches to pulse measurement, especially using pulse diagnostic devices able to categorize pulse patterns by analyzing pulse wave variation [3], quantification of left/right cun, guan, and chi pulse sections [4], and characteristics of pulse waves in specific disorders [5–9]. Among these, some studies have investigated pulse characteristics associated with gynecological diseases, including studies on aortic pulse wave analysis (PWA) in healthy premenopausal women [10] and on the association between pulse wave velocity, arteriosclerosis, and blood pressure in cases of premenstrual syndrome [11]. With regard to pulse characteristics associated with menstruation, immediately before menstruation begins, the pulse appears to be slippery rapid or string-like rapid, and the cun (inch), guan (bar), and chi (cubit) sections of the right wrist pulse are relatively clear, whereas when menstruation starts, the pulse becomes relaxed, with the chi pulse section becoming weaker [12, 13]. Some of the various types of menstrual pulses can be described as follows: a pulse coming from qi stagnation blood stasis is a string-like rough pulse; a pulse coming from cold damp stagnation is a sunken tight pulse; a pulse coming from deficiency of qi and blood is a fine weak pulse; a pulse coming from yin deficiency of liver and kidney is a sunken fine weak pulse, with the chi pulse being weak on both sides [14].

According to Chan's* Yixue rumen* (Introduction to medicine), “Even when the menstrual period is sometimes early or late, or the bleeding volume is sometimes great or little, or a month is missed, if the pulse is normal, there is no disease”; and “Even when the pulse around menstruation is normal and the cun and guan are soft, if the chi pulse is expired, then uterine pain may occur [15].” This suggests that the manifestation patterns of the cun, guan, and chi pulse sections can have greater diagnostic value than the actual clinical features of a patient's menstruation.

Research on menstrual pulse characteristics has included studies on the effect of different phases of the menstrual cycle on the reflection index, stiffness index, and pulse wave velocity in healthy subjects [16], the influence of the menstrual cycle on pulse pressure waveforms measured from the radial artery in biphasic healthy women [17], the influence of the menstrual cycle on QT interval dynamics [18], and assessment of cardiovascular autonomic functions and baroreceptor reactivity in women with premenstrual syndrome [19]. Lee et al. [20] conducted a study on variation in menstrual pulse waves compared to nonmenstrual pulse waves but were unable to concretely determine their characteristics. The present study constitutes basic research aimed at securing objective diagnostic indicators associated with a specific disorder. Using a pulse diagnostic device, we performed a comparative analysis of pulse wave variation in an experimental group with menstrual pain and a control group with little or no menstrual pain, attempting to take measurements during both the nonmenstrual and menstrual periods. As a result, we were able to obtain basic data confirming that pulse wave variation can provide important information in the diagnosis of menstrual pain.

## 2. Methods

### 2.1. Research Design

The subjects in this study were women aged between 19 and 30 years. This study measured and compared variation in the pulse waves of each group during the nonmenstrual and menstrual periods and performed a comparative analysis of pulse wave variation observed in the two groups during these times.

The criteria for participation in each group and the measurement periods during the menstrual cycle were as follows. First, the experimental group was composed of subjects who experience discomfort in daily life or interpersonal activities caused by menstrual pain, with scores of 4 or above on the measure of menstrual pain (MMP) [21]. The control group was composed of subjects reporting little or no discomfort in daily life or interpersonal activities caused by menstrual pain, with scores of 3 or below on the MMP [21]. For measurement purposes, the nonmenstrual period was considered to be 7–10 days after the last day of menstruation, while the menstrual period measurements were taken within 2-3 days following the first day of menstruation, when menstrual pain is at its peak.

### 2.2. Subjects

The subjects in this study were women residing in the city of “B,” Republic of Korea, who fully understood the purpose of the study and gave their full consent to participate (CRIS registration number: KCT0001929). This study was conducted as a pilot study on pulse information as part of a broader clinical research project involving menstrual pain for which information was collected on patients' physical form, complexion color, pulse waves, and symptoms.

#### 2.2.1. Number of Subjects

A total of 550 subjects were recruited, of whom 9 were excluded from analysis, based on the exclusion criteria. There were 329 subjects in the experimental group and 212 subjects in the control group.

#### 2.2.2. Inclusion and Exclusion Criteria for Subjects

Inclusion criteria for all subjects included sex and age (females aged 18 years or above) and length of menstrual cycle (21–39 days). For the experimental group, inclusion required reporting discomfort in daily life or interpersonal activities from menstrual pain (score of 4 or above on the MMP), while inclusion in the control group required reporting little or no discomfort in daily life or interpersonal activities from menstrual pain (score of 3 or below on the MMP). Exclusion criteria included menstrual cycle length of less than 20 days or more than 40 days, premenopausal symptoms (irregular menstruation and rosacea), polycystic ovarian syndrome (PCOS), sterility, history of cancer within the previous 5 years, severe diseases with the potential to affect signs and symptoms (cardiovascular disease, renal disease, diabetes, anemia, resistant hypertension, active liver or gallbladder disease, hyperthyroidism or hypothyroidism, psychiatric disorders or antidepressant use,* sanhupung* [postpartum syndrome], or musculoskeletal disease beyond a moderate level), or otherwise being assessed by the investigators to be either mentally or physically unsuitable to participate in the study.

### 2.3. Pulse Wave Measurement Device and Method

#### 2.3.1. Measuring Device

**Figure 1:**
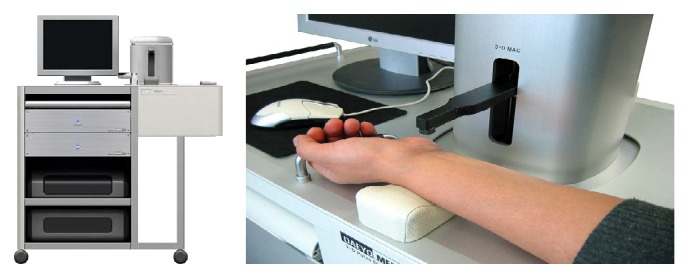
3-Dimensional pulse waveform analyzer (3-D MAC).

**Figure 2:**
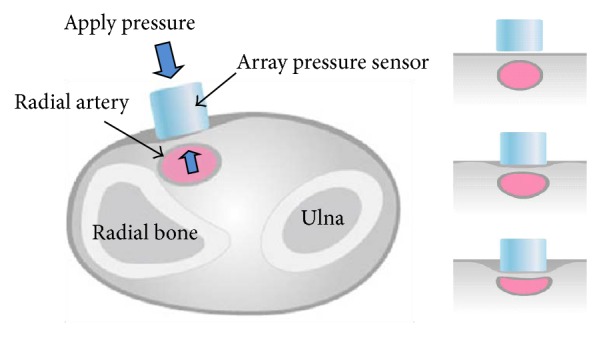
Cross section of radial artery measurement.

Subjects' pulse waves were measured using a 3-dimensional pulse waveform analyzer (3-D MAC: Daeyomedi Co., Korea, KFDA approval number: 05-178, 2005), a multichannel array piezoresistive pressure sensor that automatically checks the exact location of the radial artery and uses applanation tonometry to detect 5 different applied pressure steps [22, 23] (Figures 1 and 2). Through multichannel pressure signals, the 3-D MAC pulse analyzer enables objective, more precise measurement of 3-dimensional pulse volume, defined as pulse energy. Its motorized movements correspond to the traditional palpation positions of cun, guan, and chi, from which it acquires pulse wave data. This method differs from electrocardiographic measurements, which target the heart's electrical activity [24].

#### 2.3.2. Method

All measurements were taken at Dong-Eui Medical Center in Busan, Republic of Korea, between November 2014 and July 2015. The pulse wave measurement method was as follows. First, each subject filled out a menstrual cycle questionnaire and a general questionnaire and was given time to relax for 10 minutes before the measurement. In the case of subjects measured in both the menstrual and nonmenstrual periods, the measurements were taken at the same time of day. To measure the pulse waves of the left and right wrists in turn from the guan pulse spot at a point proximal to the radial styloid process, the subjects were seated upright in a comfortable position, resting one forearm at a time on the pulse diagnostic device. Starting with guan and moving in the direction of chi and cun, the device's sensor applied varying pressures to measure pulse waveforms at 5 separate pressure steps for 5 seconds each.

### 2.4. Data Analysis Methods

Pulse wave analysis was performed with the applanation tonometry method. In order to analyze the collected parameters, we used SAS version 9.4. The general characteristics of the subjects were analyzed through analysis of means. A paired-sample *t*-test was applied to analyze the mean differences in each group's pulses during the menstrual and nonmenstrual periods, while an independent sample *t*-test was conducted to compare the control group and experimental group during the two respective periods. The level of statistical significance was set at *p* ≤ 0.05.

## 3. Results

### 3.1. General Characteristics

The general characteristics of the subjects in the experimental and control groups are presented in Table 1. No statistically significant differences existed between the means of the two groups' height, weight, body mass index (BMI), and heart rate (HR). However, there was a slightly significant difference in age. The mean ages of the experimental and control group were 22.16 and 22.52, respectively.

### 3.2. Pulse Wave Factor Characteristics

#### 3.2.1. Comparison of Pulse Waves of the Experimental and Control Groups during the Nonmenstrual Period

The pulse wave characteristics of the experimental and control groups measured during the nonmenstrual period are presented in Table 2. With respect to the pulse waves measured from the left radial artery, the mean value of the radial augmentation index (RAI) (*t* = 1.96; *p* = 0.050) of the experimental group was significantly higher compared to control group, while no other pulse wave factors presented any statistically significant differences between the two groups. In the case of the right radial artery, the experimental group had lower values than the control group for pulse wave energy (*t* = −2.32; *p* = 0.021), time to first peak from baseline (*T*1) (*t* = −2.11; *p* = 0.035), and mean energy per minute (*E*/min) (*t* = −2.01; *p* = 0.045), while no other pulse wave factors presented any significant differences between the two groups.

#### 3.2.2. Comparison of Pulse Waves of the Experimental and Control Groups during the Menstrual Period

The characteristics of the pulse waves of the experimental and the control groups measured during the menstrual period are presented in Table 3. In a comparison of pulse waves measured from the left radial artery, the experimental group showed significantly lower values than the control group for the cardiac diastolic phase (*T* − *T*4) (*t* = −2.14; *p* = 0.034) and area of pulse wave during the diastolic phase (*A*_d_) (*t* = −1.97; *p* = 0.049), while it had significantly higher values for the area of the pulse wave during the systolic phase (*A*_S_) (*t* = 1.97; *p* = 0.049), the ratio of the systolic phase to the total pulse time (*T*4/*T*) (*t* = 2.56; *p* = 0.011), and the ratio of the systolic phase to the diastole (*T*4/(*T* − *T*4)) (*t* = 2.44; *p* = 0.015). However, no other pulse wave factors presented any statistically significant differences between the two groups.

With respect to the right radial artery, no pulse wave factors presented any statistically significant differences between the two groups.

#### 3.2.3. Comparison of Pulse Wave Variation in Experimental Group and Control Group during Menstrual and Nonmenstrual Periods

The comparison of pulse wave variation observed during the menstrual and nonmenstrual periods in the experimental group and the control group is presented in Table 4. A paired-sample *t*-test was applied to analyze the mean differences in each group's pulse waves during the menstrual and nonmenstrual periods, while an independent sample *t*-test was conducted to compare the control group and experimental group during the two respective periods. With respect to the pulse waves measured from the left radial artery, the experimental group showed significantly less variation in *T* − *T*4 (*t* = −2.45; *p* = 0.015) values, while the experimental group showed greater variation in *T*4/*T* (*t* = 2.85; *p* = 0.005) and *T*4/(*T* − *T*4) (*t* = 2.46; *p* = 0.014) values. However, no other pulse wave factors presented any statistically significant differences between the two groups.

With respect to the pulse waves measured from the right radial artery, the experimental group showed significantly greater variation in pulse wave energy (*t* = 2.21; *p* = 0.028), vascular resistance (indicated by *H*4) (*t* = 2.05; *p* = 0.041), left ventricular systole (*T*4) (*t* = 2.48; *p* = 0.014), and representative pulse wave area (*A*_p_) (*t* = 2.25; *p* = 0.026). However, no other pulse wave factors presented any statistically significant differences between the two groups.

## 4. Discussion

The pulse is one of the major diagnostic methods clinically applied in traditional Korean medicine, and, in recent years, diverse scientific approaches to pulse diagnosis have been explored [25, 26]. Besides hormonal changes during the menstrual period, prostaglandins are excreted. The prostaglandin PGF2*α* in particular causes intense vasoconstriction, affecting vessel wall tension and producing changes in the pulse wave [27]. This study aimed to investigate pulse wave factors associated with menstrual pain, using a pulse diagnosis device to measure the pulse waves of female subjects with menstrual pain, alongside a comparison group with little or no menstrual pain, during both the menstrual and nonmenstrual periods. A comparative analysis of changes in the two groups' pulse wave factors during each of the two periods was then conducted.

Regarding radial artery pulse waves in the study of primary dysmenorrhea, Chen et al. (2015) chose to investigate only the left guan floating pulse, explaining that gynecologic symptoms are more clearly manifested in the left wrist pulse. However, in general, the blood pressure on the right side is higher than that on the left side [28]. The theoretical background for this difference is found in the theories of left blood, right qi, and organ localization. Accordingly, in this study, we chose to perform pulse wave analysis for both sides.

*During the Nonmenstrual Period*. In the comparison of pulse waves measured from the left radial artery in each group during the nonmenstrual period, the RAI value of the experimental group was significantly higher compared to the control group (*t* = 1.96; *p* = 0.050). The RAI (radial augmentation index) is calculated as the ratio of the height of the aortic valley (*H*2) to the height of the main peak (*H*1) or as the ratio of the height of the aortic peak (*H*3) to the height of the main peak (*H*1). As an indicator of arterial compliance, it serves as an index of cardiovascular elasticity [8, 23]. In general, RAI values increase over the long term as people age. They also increase as blood vessels stiffen or tense. As such, RAI is used to assess blood vessel tension and stiffness as well as peripheral circulation. The RAI values of the experimental group were significantly higher compared to the control group, which suggests that the blood vessels of women with menstrual pain have greater stiffness and tension compared to women with little or no menstrual pain (see Table 2).

In pulse waves measured from the right radial artery during the nonmenstrual period, the experimental group showed significantly lower values than the control group for pulse wave energy (*t* = −2.32; *p* = 0.021) and time to first peak from baseline (*T*1) (*t* = −2.11; *p* = 0.035). Pulse wave energy is the integral value of all measured sensor signals obtained from a pulsation area corresponding to the fingertip of the pulse taker. The intensity of the pulsation is calculated based on its 3-dimensional volume, which can be utilized as an objective pulse wave factor indicating strong or weak pulse in traditional pulse diagnosis [22]. The fact that the values for pulse wave energy and *T*1 were significantly lower in the experimental group than in the control group suggests that women with menstrual pain lack pulsatile force, which results in shortened systolic phases compared with women with little or no menstrual pain (see Table 2). To sum up, compared with women with little or no menstrual pain, women with menstrual pain display low blood vessel elasticity and low pulsatile force, leading to a shortened diastolic phase in the pulse wave cycle and shortened time to first peak from baseline.

*During the Menstrual Period*. In the comparison of left radial artery pulse waves in the experimental and control groups during the menstrual period, the cardiac diastolic phase (*T* − *T*4) (*t* = −2.14; *p* = 0.034) and the ratio of the diastolic phase to the pulse waveform area (*A*_d_) (*t* = −1.97; *p* = 0.049) were significantly lower in the experimental group than in the control group. The experimental group had significantly higher values for the ratio of the systolic phase to the pulse waveform area (*A*_s_) (*t* = 1.97; *p* = 0.049), the ratio of the systolic phase to the total pulse time (*T*4/*T*) (*t* = 2.56; *p* = 0.011), and the ratio of the systolic phase to the diastole *T*4/(*T* − *T*4) (*t* = 2.44; *p* = 0.015). This can be seen as the result of an increase in the systolic phase and a decrease in the diastolic phase in the cardiac cycle during the menstrual period. However, in the right radial artery, no pulse wave factors presented any statistically significant differences between the two groups (see Table 3). In conclusion, menstrual pulse waves observed in women with menstrual pain were found to be characterized by a relatively prolonged systolic phase and shortened diastolic phase, which is presumed to correlate with increased cardiac output or cardiac loading, thus resulting in the heart pumping more blood out to the peripheral circulation.

With respect to the pulse waves measured from left radial artery during the menstrual period, changes in the experimental group's values for *T* − *T*4 (*t* = −2.45; *p* = 0.015) were significantly lower compared to the control group, while changes in the values for *T*4/*T* (*t* = 2.85; *p* = 0.005) and *T*4/(*T* − *T*4) (*t* = 2.46; *p* = 0.014) were greater, resulting in the amplification of the pulse wave features of a prolonged systolic phase and shortened diastolic phase during menstruation, compared with the nonmenstrual period. With respect to the pulse waves measured from the right radial artery, the experimental group had significantly higher values than the control group for pulse wave energy (*t* = 2.21; *p* = 0.028), blood vessel resistance (*H*4) (*t* = 2.05; *p* = 0.041), left ventricular systole (*T*4) (*t* = 2.48; *p* = 0.014), and representative pulse wave area (*A*_p_) (*t* = 2.25; *p* = 0.026) (see Table 4). The increases in pulse energy, representative pulse wave area, ventricular systole, and blood vessel resistance can all be considered to be related to the features of the menstrual bleeding period.

Therefore, women with menstrual pain are likely to have decreased blood vessel elasticity, low pulsatile force, reduced diastolic phase in the pulse wave cycle, and shorter time to first peak from baseline during the nonmenstrual period, as well as prolonged systolic phase and shortened diastolic phase during the menstrual period, leading to increased cardiac output or cardiac loading, which increases the likelihood of greater bleeding volume compared to women without menstrual pain.

Prior research targeting normal women of childbearing age has found decreased blood pressure in the late follicular phase and decreased vessel stiffness [16, 29, 30]. However, this study found that the pulse waves of the menstrual pain group were the exact opposite of the normal comparison group.

The higher RAI during the nonmenstrual period signifies increased stiffness or decreased elasticity in addition to increased blood pressure and blood viscosity. Furthermore, the change toward lower pulsatile force and shorter time to first peak from baseline can cause blood circulation problems associated with blood stagnation, which is considered the major cause of menstrual pain in traditional Korean medicine [31].

An increase in left ventricular systole (*T*4), unaccompanied by noticeable change in blood pressure or heart rate, signifies abnormality of the aortic valve or increased cardiac preload [32]. During the menstrual period, an increase in LVET suggests increased left ventricular preload, namely, an increase in blood circulation. This is a likely explanation for why menstrual pain is often accompanied by menorrhea.

In other words, tension of the blood vessels during the nonmenstrual period is related to blood stasis and hypertension of the autonomic nervous system. Further, prolongation of the systole during the menstrual period correlates with increased menstrual pain and hemodynamic changes.

In women with menstrual pain, pulse waves in the menstrual period are prominently marked by prolonged systolic and shortened diastolic phases, compared with the nonmenstrual period. In addition, during the menstrual period, increases are seen in pulse wave energy, representative pulse wave area, length of the systolic phase, and blood vessel resistance. These findings further suggest that future research is needed to investigate the mechanism behind the menstrual pulse wave which is a slippery rapid or string-like rapid pulse [12, 13].

The following elements should be considered when interpreting the results of this study. The study did not include menstrual pain in all ages, and as the study involved relatively young women between the ages of 19 and 30 years, the subjects could be expected to show a lower rate of underlying diseases. In addition, it could be considered a limitation of the study that less than half of the subjects had their pulse waves measured during both the menstrual and nonmenstrual periods.

## 5. Conclusion

This study derived the following results. During the nonmenstrual period, women with menstrual pain display low blood vessel elasticity, low pulsatile force, and shortened time to first peak from baseline. During the menstrual period, compared with the nonmenstrual period, the pulse waves of women with menstrual pain are prominently marked by prolonged systolic and shortened diastolic phases. The pulse waves also show increases in pulse wave energy, representative pulse wave area, systolic phase, and blood vessel resistance. These indicators can be proposed as objective indices to distinguish the pulse wave-related pathological states of menstrual pain.

## Figures and Tables

**Table 1:** General characteristics of the subjects.

Group	Experimental group (*n* = 329)	Control group (*n* = 212)	*t*-value	*p* value
Variable	Mean ± SD	Mean ± SD		
Age	22.16 ± 1.97	22.52 ± 2.16	−2.01	0.045
Height	160.95 ± 4.97	161.56 ± 4.67	−1.41	0.160
Weight	53.98 ± 7.52	55.01 ± 7.99	−1.52	0.129
BMI	20.84 ± 2.79	21.04 ± 2.79	−0.85	0.396
Heart rate	78.07 ± 10.17	77.50 ± 10.87	0.61	0.543
MMP	6.72 ± 1.17	1.25 ± 1.38	47.78	<0.001

BMI: body mass index.

MMP: measure of menstrual pain.

**Table 2:** Pulse waves of the experimental and control groups during the nonmenstrual period.

Variables	Left radial artery				Right radial artery			
	Experimental group Mean ± SD	Control group Mean ± SD	*t*-value	*p* value	Experimental group Mean ± SD	Control group Mean ± SD	*t*-value	*p* value
Energy	390.371 ± 182.911	403.244 ± 192.204	−0.77	0.442	314.971 ± 183.084	354.765 ± 201.115	−2.32	0.021
*H*4	40.925 ± 16.464	40.619 ± 16.959	0.21	0.837	32.242 ± 14.724	32.873 ± 15.984	−0.46	0.646
*T*1	0.107 ± 0.009	0.107 ± 0.009	0.66	0.507	0.111 ± 0.010	0.113 ± 0.010	−2.11	0.035
*T*4	0.319 ± 0.033	0.318 ± 0.027	0.60	0.551	0.310 ± 0.030	0.314 ± 0.030	−1.64	0.101
*T* − *T*4	0.467 ± 0.103	0.474 ± 0.108	−0.73	0.463	0.450 ± 0.093	0.450 ± 0.097	0.00	1.000
*A*_p_	7901.274 ± 2949.925	8065.463 ± 2933.796	−0.62	0.534	6591.526 ± 2771.091	7004.789 ± 3045.965	−1.59	0.112
*A*_s_	71.827 ± 6.808	71.332 ± 6.572	0.82	0.411	70.897 ± 6.607	71.368 ± 6.738	−0.78	0.433
*A*_d_	28.173 ± 6.808	28.668 ± 6.572	−0.82	0.411	29.103 ± 6.607	28.632 ± 6.738	0.78	0.433
RAI	60.784 ± 15.347	58.176 ± 13.967	1.96	0.050	55.138 ± 14.801	54.661 ± 15.042	0.36	0.722
*E*/min	30518.311 ± 15053.620	31116.693 ± 15348.294	−0.44	0.660	25274.641 ± 15046.942	28016.549 ± 15380.574	−2.01	0.045
*T*4/*T*	0.411 ± 0.047	0.407 ± 0.047	0.96	0.340	0.413 ± 0.047	0.417 ± 0.048	−0.94	0.345
*T*4/(*T* − *T*4)	0.710 ± 0.139	0.698 ± 0.136	0.97	0.334	0.713 ± 0.141	0.726 ± 0.144	−0.97	0.334

Energy: 3-dimensional pulse volume from multipoint array pressure sensor. Pulse volume can be calculated by integration of pulse amplitude envelope.

*H*4: amplitude of incisura is affected by amplitude of reflected wave.

*T*1: time to first peak from baseline correlates with vessel compliance.

*T*4: duration of left ventricular systole.

*T* − *T*4 : left ventricle diastolic phase; T is equivalent to one pulse period.

*A*_p_: pulse wave area.

*A*_s_: ratio of systolic phase to pulse wave area.

*A*_d_: ratio of diastolic phase to pulse wave area.

RAI: radial augmentation index, which is calculated as *H*3 : *H*1 (ratio of height of the aortic peak to the height of the main peak), related to vessel's stiffness and aging.

*E*/min: mean energy per minute.

*T*4/*T*: ratio of systolic phase to total pulse period.

*T*4/(*T* − *T*4) : ratio of systolic phase to diastolic phase.

Note: increased duration of systolic phase may indicate increased stroke volume or cardiac loading.

**Table 3:** Pulse waves of the experimental and control groups during the menstrual period.

Variables	Left radial artery				Right radial artery			
	Experimental group Mean ± SD	Control group Mean ± SD	*t*-value	*p* value	Experimental group Mean ± SD	Control group Mean ± SD	*t*-value	*p* value
Energy	395.078 ± 195.431	393.277 ± 188.691	0.08	0.937	317.253 ± 184.568	326.358 ± 194.467	−0.40	0.688
*H*4	41.647 ± 16.551	42.055 ± 17.667	−0.20	0.840	33.003 ± 15.682	31.632 ± 16.157	0.72	0.473
*T*1	0.108 ± 0.011	0.109 ± 0.011	−0.92	0.360	0.112 ± 0.013	0.111 ± 0.011	0.53	0.598
*T*4	0.326 ± 0.030	0.319 ± 0.031	1.94	0.054	0.316 ± 0.030	0.312 ± 0.039	0.90	0.367
*T* − *T*4	0.442 ± 0.093	0.475 ± 0.148	−2.14	0.034	0.434 ± 0.087	0.442 ± 0.117	−0.62	0.536
*A*_p_	8043.088 ± 3295.989	8275.545 ± 3379.887	−0.59	0.557	6606.237 ± 2970.010	6645.138 ± 2972.616	−0.11	0.913
*A*_s_	73.202 ± 6.326	71.357 ± 8.634	1.97	0.049	71.837 ± 6.991	72.229 ± 6.778	−0.47	0.637
*A*_d_	26.798 ± 6.326	28.643 ± 8.634	−1.97	0.049	28.163 ± 6.991	27.771 ± 6.778	0.47	0.637
RAI	61.391 ± 14.552	62.742 ± 13.622	−0.80	0.425	57.356 ± 15.316	55.543 ± 15.210	0.99	0.324
*E*/min	31113.389 ± 15765.610	31277.134 ± 16374.795	−0.09	0.931	25810.805 ± 15133.580	26862.294 ± 16885.758	−0.55	0.580
*T*4/*T*	0.430 ± 0.050	0.412 ± 0.062	2.56	0.011	0.426 ± 0.050	0.421 ± 0.052	0.88	0.379
*T*4/(*T* − *T*4)	0.769 ± 0.162	0.720 ± 0.176	2.44	0.015	0.755 ± 0.155	0.740 ± 0.159	0.83	0.406

See Table 2 for explanation of variables.

**Table 4:** Comparison of pulse wave variation in menstrual and nonmenstrual periods.

Variables	Left radial artery				Right radial artery			
	Experimental group Mean ± SD	Control group Mean ± SD	*t*-value	*p* value	Experimental group Mean ± SD	Control group Mean ± SD	*t*-value	*p* value
Energy	1.250 ± 215.386	−33.275 ± 235.934	1.29	0.200	1.094 ± 210.381	−55.481 ± 207.855	2.21	0.028
*H*4	0.653 ± 19.981	−0.778 ± 22.635	0.57	0.572	1.540 ± 18.280	−2.902 ± 16.585	2.05	0.041
*T*1	0.000 ± 0.014	0.000 ± 0.012	−0.24	0.814	0.000 ± 0.014	−0.003 ± 0.012	1.71	0.088
*T*4	0.006 ± 0.039	−0.001 ± 0.031	1.68	0.095	0.006 ± 0.035	0.005 ± 0.038	2.48	0.014
*T* − *T*4	−0.023 ± 0.130	0.017 ± 0.148	−2.45	0.015	−0.006 ± 0.100	−0.005 ± 0.100	−0.12	0.908
*A*_p_	123.309 ± 3785.597	−161.853 ± 4360.963	0.59	0.555	114.514 ± 3448.130	−782.094 ± 2922.997	2.25	0.026
*A*_s_	1.197 ± 8.056	−0.486 ± 8.705	1.68	0.093	0.448 ± 7.933	0.245 ± 7.792	0.21	0.834
*A*_d_	−1.197 ± 8.056	0.486 ± 8.705	−1.68	0.093	−0.448 ± 7.933	−0.245 ± 7.792	−0.21	0.834
RAI	0.355 ± 18.014	3.299 ± 15.837	−1.42	0.157	2.390 ± 18.039	0.230 ± 17.095	1.00	0.319
*E*/min	19.324 ± 17625.167	−2068.532 ± 18936.432	0.96	0.339	186.740 ± 17371.810	−3431.774 ± 18894.926	1.65	0.100
*T*4/*T*	0.016 ± 0.060	−0.004 ± 0.057	2.85	0.005	0.008 ± 0.055	0.000 ± 0.046	1.34	0.181
*T*4/(*T* − *T*4)	0.049 ± 0.184	−0.003 ± 0.162	2.46	0.014	0.026 ± 0.177	0.001 ± 0.145	1.26	0.208

See Table 2 for explanation of variables.
